# Treatment of pruritus in primary sclerosing cholangitis: Analysis of the consortium for autoimmune liver disease registry

**DOI:** 10.1097/HC9.0000000000000703

**Published:** 2025-05-06

**Authors:** Richard Dean, Maryam Yazdanfar, Joseph Zepeda, Cynthia Levy, Craig Lammert, Daniel Pratt, Stuart C. Gordon, Lisa Forman, David N. Assis, Ashleigh McGirr, Megan McLaughlin, Sumanta Mukherjee, Usha Gungabissoon, Christopher L. Bowlus

**Affiliations:** 1 Division of Gastroenterology and Hepatology, University of California Davis, Sacramento, California, USA; 2 Schiff Center of Liver Disease and Division of Gastroenterology and Hepatology, University of Miami, Miami, Florida, USA; 3 Indiana University, Indianapolis, Indiana, USA; 4 Massachussetts General Hospital, Boston, Massachussetts, USA; 5 Department of Gastroenterology and Hepatology, Henry Ford Health, Detroit Michigan, USA; 6 Wayne State University School of Medicine, Detroit Michigan, USA; 7 Univeristy of Colorado, Denver, Colorado, USA; 8 Section of Digestive Diseases, Yale School of Medicine, New Haven, Connecticut, USA; 9 GSK, Mississauga, Ontario, Canada; 10 GSK, Collegeville, Pennsylvania, USA; 11 GSK, London, UK

**Keywords:** cholestasis, symptoms, treatment

## Abstract

**Background:**

Cholestasis from primary sclerosing cholangitis (PSC) frequently causes pruritus. However, the prevalence of pruritus and its management have not been well studied. Investigating the Cholestatic Pruritus of Primary Sclerosing Cholangitis (ItCh-PSC) includes a retrospective medical record review to determine the prevalence, severity, and treatment patterns of pruritus.

**Methods:**

Data was collected at 5 academic medical centers in the United States. Medical records were searched for the terms “itch” and “pruritus” and data abstracted related to itch severity, number of encounters, and treatment.

**Results:**

Among 724 patients with PSC, 359 (50%) of patients had a documented history of pruritus, including 40%, 39%, and 21% with mild, moderate, or severe itch. Itch was less common in those with small ducts compared to large duct PSC (*p*=0.02) and more frequent in those of Hispanic versus non-Hispanic ethnicity (*p*=0.001). Compared to patients with mild itch, patients with moderate or severe itch were younger, and had more elevated liver biochemistries, more encounters with itch, and more frequently prescribed 2 or more anti-pruritic medications. Bile acid–binding resins were prescribed in 36%, hydroxyzine in 23%, rifampin in 11%, and fenofibrate in 4% of patients with any itch. The prevalence and severity of pruritus were not affected by cirrhosis, hepatic decompensation, or inflammatory bowel disease.

**Conclusion:**

Itch is common in patients with PSC and is often associated with multiple prescriptions of antipruritic agents. Effective treatments for pruritus in patients with PSC remain an unmet need.

## Graphical abstract

**Figure:**
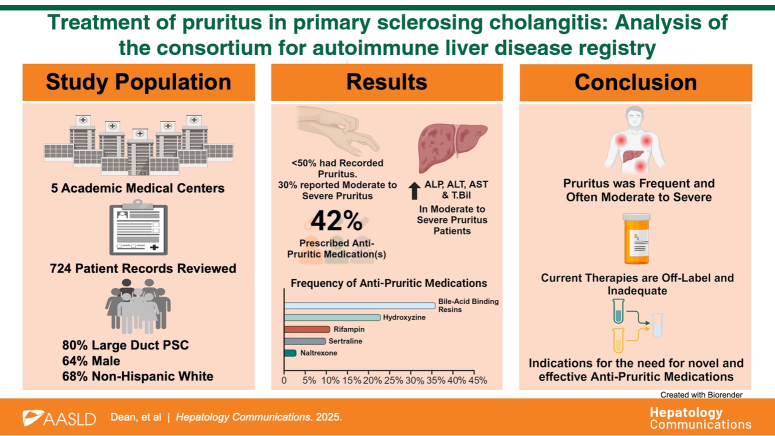

## INTRODUCTION

Primary sclerosing cholangitis (PSC) is a chronic liver disease characterized by inflammation and fibrosis of the bile ducts, leading to strictures and obstruction of bile flow.^1^ PSC is a rare condition, with an estimated median prevalence of 6.0–7.4 cases per 100,000 people.^2^ While some risk factors for PSC have been identified, specifically inflammatory bowel disease (IBD), the underlying mechanisms of disease development and progression are still largely unknown.^3^ Currently, there are no approved therapies for PSC or its related symptoms. PSC has been associated with impaired quality of life and a higher frequency of depression,^4^ and like other cholestatic liver diseases, pruritus is a common and distressing symptom in patients with PSC.

The exact pathophysiology of pruritus is not fully understood but is thought to be related to the accumulation of pruritogens in the blood and/or skin, which activate sensory receptors and lead to the central sensation of itch.^5^^,^^6^ Bile acids have long been suspected as the pruritogen, but others, including lysophosphatidic acid and IL-31, have also been implicated.^7^^,^^8^ Sensory receptors, including histamine receptors, opioid receptors, and G protein-coupled receptors (such as MAS-related G protein-coupled receptor A3), have also been suggested to mediate the itch signal.^9^

Current guidelines suggest therapies for pruritus target serum bile acids, histamines, and opioid receptors, but have limited effectiveness.^10^ Bile acid sequestrants, such as cholestyramine, are commonly used for the first-line treatment of pruritus in PSC and function to reduce serum bile acid levels by enhancing their excretion in stool.^1^^,^^11^^,^^12^ Other agents targeting bile acids include rifampin, a pregnant × receptor agonist, and peroxisome proliferator-activated receptor (PPAR) agonists like bezafibrate and fenofibrate, that inhibit bile acid synthesis.^13^^,^^14^ Targeting of the pruritogen receptor includes antihistamines, selective serotonin reuptake inhibitors, and opioid receptor antagonists, which have shown limited effectiveness.^15^^,^^16^

Although the frequency, severity, and impact of pruritus have been well documented in primary biliary cholangitis (PBC), it has not been well studied in PSC. The prevalence of pruritus reported in retrospective case series has ranged from 25% to 69% but likely underestimates the true prevalence of pruritus among patients with PSC.^17^^–^^21^ Among 811 PSC patients enrolled in an online registry, 66.4% of United States and 58.9% of non-US participants reported itching of any severity with a mean severity of itching of 2.7±2.9 and 2.0±2.4 on a 10-point scale among US and non-US participants, respectively.^22^ PSC has also been associated with impaired quality of life and a higher frequency of depression.^4^^,^^22^

Investigating the Cholestatic Pruritus of Primary Sclerosing Cholangitis (ItCh-PSC) is a 3-part study including a retrospective medical record review, a cross-sectional cohort study, and a prospective observational study. Herein, we report the results of the first part of ItCh-PSC, a detailed, retrospective analysis of pruritus and its treatment in a large, multicenter cohort of patients with PSC.

## METHODS

### Study population

The Consortium for Autoimmune Liver Diseases (CALiD) is a retrospective-prospective research registry involving 18 centers throughout North America; study methods have been previously described.^23^ All patients with PSC from 5 of the CALiD sites were included in the current study. The sample includes all patients over the age of 18 with PSC alive in January 2008 or later, without a liver transplant prior to January 2008, and with at least 1 encounter after January 2008 at each center. Diagnostic criteria for PSC were consistent with current recommendations.^1^^,^^11^ Exclusion criteria included the presence of other concomitant liver diseases, death, or liver transplantation prior to 2008 or the absence of data in the medical record after 2008. Data was collected from visits between January 2008 and November 2022.

### Data collection

Medical records were electronically searched for the terms “itch” and “pruritus” to identify all patient encounters with a provider notation related to pruritus. When multiple notes were present in a 6-month period, only the note describing the most severe itch was included for analysis; otherwise, all encounters were recorded and abstracted. Data on the severity, frequency, and change in itch, as well as planned interventions for itch described in the encounter by the healthcare provider, were abstracted. Severity was classified as absent, mild, moderate, or severe, based on the descriptors found in the medical record. If there were multiple descriptors in a note, the most severe was used for grading severity. Confounding dermatologic conditions, including psoriasis, atopic dermatitis, and eczema, were noted. Medications of interest and the indication, dosage, frequency of administration, and start date were recorded for antipruritic medications, as well as ursodeoxycholic acid (UDCA) and medications for the treatment of IBD. Demographic data, type of PSC, disease duration, and baseline laboratory data from the CALiD registry were merged with the ItCh-PSC data. Cirrhosis was defined by cirrhotic morphology on imaging, liver stiffness by transient elastography (TE) >14.4 kPa or magnetic resonance elastography (MRE) >4.93 kPa, or aspartate aminotransferase to platelet ratio index (APRI) >2.

### Statistical analysis

Demographic, clinical, and laboratory characteristics for the entire cohort were summarized using descriptive statistics. Data are presented as median (IQR). Subgroup analyses were performed based on the absence or presence of at least 1 itch-related encounter of mild, moderate, or severe itch and by the severity of itch among those with at least 1 itch-related encounter. When more than 1 itch-related encounter was present, patients were categorized by the most severe itch. Chi-squared (*χ* ^2^) test of independence was used to assess categorical variables. Mann–Whitney *U* test or Kruskal–Wallis test were applied as appropriate for continuous variables. All statistical analyses were conducted using SAS statistical software (SAS Institute) version 9.4. A *p* value (2-tailed test) of 0.05 was used as the threshold for statistical significance.

### Ethical considerations

All research was approved by the institutional review board of each participating site and was conducted in accordance with the Declaration of Helsinki and Istanbul guidelines. All patients enrolled in the prospective arm of CALiD or ItCh-PSC provide informed consent. For patients enrolled only in retrospective arms, only de-identified data were collected, and the requirement for informed consent was waived at each site.

## RESULTS

### Frequency and severity of itch

A total of 724 patients from 6 sites were included in the study; 372 (51%) had at least 1 itch-related encounter in their medical record (Table 1). After excluding 13 patients whose encounters noted the absence of itch, 359 (50%) of patients had documented pruritus, of which 19 patients (2%) were identified with potentially confounding dermatologic conditions.

**TABLE 1:** Demographic and clinical characteristics of the ItCh-PSC cohort and comparison of those with and without the documented itch

	Overall (n=724)	No itch (n=365)	Documented itch (n=359)	*p*
Age at time of enrollment (n=710)	49.0 (36.0-61.0)	50.0 (35.0–61.0)	49.0 (36.0–60.0)	0.69
Age at diagnosis^a^ (n=583)	41.0 (30.0–54.0)	42.0 (30.0–55.0)	40.0 (30.0–53.0)	0.38
Age at diagnosis^b^ (n=677)	39.0 (27.0–51.0)	39.0 (27.0–51.0)	38.0 (27.0–51.0)	0.80
Sex (male) (n=705), n (%)	458 (63)	240 (66)	218 (61)	0.37
Race (n=724), n (%)				
White	597 (82)	310 (85)	287 (80)	0.23
Black/African American	74 (10)	29 (8)	45 (13)	
Other	28 (4)	14 (4)	14 (4)	
Unknown/missing	25 (3)	12 (3)	13 (4)	
Ethnicity (n=724), n (%)				
Hispanic	54 (7)	16 (4)	38 (11)	0.001
Non-Hispanic	635 (88)	325 (89)	310 (86)	
Unknown/missing	35 (5)	24 (7)	11 (3)	
PSC type, n (%)				
Large duct	578 (80)	290 (79)	288 (80)	0.02
PSC/AIH overlap	50 (7)	21 (6)	29 (8)	
Small duct PSC	60 (8)	40 (11)	20 (6)	
Unknown/missing	36 (5)	14 (4)	22 (6)	
Cirrhosis,^c^ n (%)	169 (23)	84 (23)	85 (24)	0.23
Hepatic decompensation,^d^ n (%)	95 (13)	42 (12)	53 (15)	0.15
IBD status, n (%)				
Ulcerative colitis	364 (50)	188 (52)	176 (49)	0.37
Crohn disease	90 (12)	43 (12)	47 (13)	
Indeterminate colitis	12 (2)	6 (2)	6 (2)	
No IBD	226 (31)	107 (29)	119 (33)	
Unknown/missing	32 (4)	21 (6)	11 (3)	
Laboratories^e^				
ALP (IU/L)	198.0 (116.0–368.0)	187.0 (116.0–365.0)	208.0 (116.0–389.0)	0.31
AST (IU/L)	45.0 (28.0–83.0)	44.0 (28.0–79.0)	48.0 (29.0–87.0)	0.26
ALT (IU/L)	49.0 (27.0–88.0)	48.0 (25.0–84.0)	51.5 (31.0–90.0)	0.08
Total bilirubin (mg/dL)	0.9 (0.6–2.0)	0.8 (0.5–2.1)	0.9 (0.6–2.0)	0.38
Hemoglobin (g/dL)	13.2 (11.6–14.4)	13.2 (11.6–14.4)	13.3 (11.8–14.4)	0.88
Platelets (×10^6^/mm^3^)	220.0 (148.0–290.0)	216.5 (145.0–280.0)	223.0 (149.0–290.0)	0.49
INR	1.1 (1.0–1.2)	1.1 (1.0–1.3)	1.0 (1.0–1.2)	0.03

^a^ Based on the first abnormal cholangiogram.

^b^ Based on the first abnormal liver biochemistry.

^c^ Cirrhosis is defined by cirrhotic morphology on imagine, TE >14.4 kPa, MRE >4.93 kPa, APRI >2, or reported cirrhosis with stage 4.

^d^ Presence of ascites, variceal hemorrhage, and/or hepatic encephalopathy.

^e^ Laboratory values at baseline.

Abbreviations: AIH, autoimmune hepatitis; APRI, aspartate aminotransferase to platelet ratio index; IBD, inflammatory bowel disease; INR, international normalized ratio; ItCh-PSC, Investigating the Cholestatic Pruritus of Primary Sclerosing Cholangitis; MRE, magnetic resonance elastography; PSC, primary sclerosing cholangitis; TE, transient elastography.

Overall, the majority of patients were White (68%) and male (64%), though Black/African Americans made up 10% of the cohort and 7% were Hispanic. The majority of patients (80%) had large duct PSC; small duct PSC and PSC with features of autoimmune hepatitis (PSC/AIH) represented 8% and 7%, respectively. Concomitant IBD was present in 67% of those with known IBD status. Cirrhosis was present in 23% of patients, and 13% had had at least 1 hepatic decompensation event at baseline. Compared to patients without documented itching, patients with itch were less likely to have small duct PSC and were disproportionately Hispanic. Otherwise, the groups with and without documented itch were similar.

**FIGURE 1:**
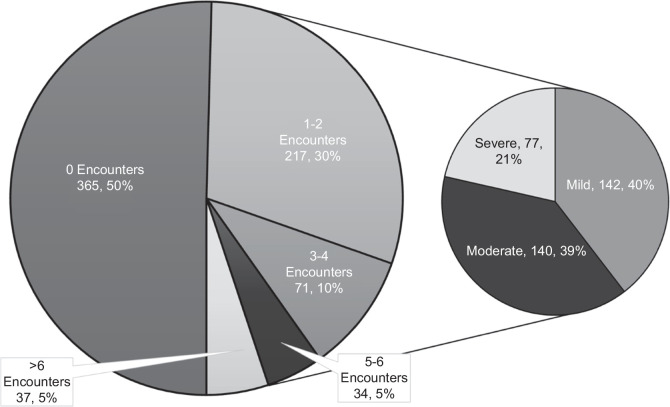
Frequency of a number of itch encounters (*left*) and distribution of itch severity among those with documented itch (*right*).

Among patients with documented itch, 217 (60%) had 1–2 itch-related medical encounters, 105 (29%) had 3–6 encounters, and 37 (10%) had >6 encounters (Figure 1). The median (IQR) itching encounters per month was 0.12 (0.07–0.27). The maximum itch severity in over half of patients was classified as moderate (39%, n=140) or severe (21%, n=77). Patients recorded with moderate-to-severe itch represented 94% of patients with >6 itch encounters; in contrast, patients with mild itch represented 55% of those with only 1–2 itch encounters (*p*<0.001).

Compared to patients with mild pruritus, patients with moderate or severe pruritus were younger at diagnosis (*p*=0.01) and had higher levels of ALP (*p*=0.01), AST (*p*=0.001), ALT (*p*=0.04), and total bilirubin (*p*=0.02) (Table 2). No associations were found between itch severity and sex, race/ethnicity, PSC type, IBD status, cirrhosis, or hepatic decompensation.

**TABLE 2:** Comparisons by maximum provider-recorded itch severity among patients with any recorded itch

	Mild, n=142	Moderate, n=140	Severe, n=77	*p*
Age at time of enrollment	52.0 (37.0–62.0)	49.0 (38.0–61.0)	43.5 (33.5–57.5)	0.08
Age at diagnosis^1^	42.0 (32.0–55.0)^a^	40.0 (29.5–54.0)^a,b^	36.0 (26.0–47.0)^b^	0.02
Age at diagnosis^2^	41.5 (32.0–54.0)	38.0 (30.0–53.0)	38.0 (27.0–47.5)	0.08
Sex (male)	89 (63%)	77 (55%)	52 (68%)	0.44
Race, n (%)				
White	119 (84)	104 (74)	64 (83)	0.47
Black/African American	13 (9)	23 (16)	9 (12)	
Other	6 (4)	6 (4)	2 (3)	
Unknown/missing	4 (3)	7 (5)	2 (3)	
Ethnicity, n (%)				
Hispanic	16 (11)	16 (11)	6 (8)	0.83
Non-Hispanic	123 (87)	119 (85)	68 (88)	
Unknown/missing	3 (2)	5 (4)	3 (4)	
PSC type, n (%)				
Large duct	120 (85)	105 (75)	63 (82)	0.35
PSC/AIH overlap	9 (6)	14 (10)	6 (8)	
Small duct PSC	6 (4)	12 (9)	2 (3)	
Unknown/missing	7 (5)	9 (6)	6 (8)	
UDCA dosage (mg/d)	1651.1±1202.1	1266.1±739.3	1292.1±677.2	0.02
Cirrhosis^3^, n (%)	33 (23)	36 (26)	16 (21)	0.88
Hepatic decompensation^4^, n (%)	21 (15)	25 (18)	7 (9)	0.47
IBD status, n (%)				
Ulcerative colitis	71 (50)	64 (46)	41 (53)	0.85
Crohn disease	18 (13)	22 (16)	7 (9)	
Indeterminate colitis	1 (1)	3 (2)	2 (3)	
No IBD	48 (34)	47 (34)	24 (31)	
Unknown/missing	4 (3)	4 (3)	3 (4)	
Laboratories				
ALP (IU/L)	171.0 (94.0–299.0)^a^	236.0 (139.0–481.0)^b^	246.0 (157.5–435.0)^a,b^	0.01
AST (IU/L)	40.0 (27.0–56.0)^a^	57.0 (35.0–108.0)^b^	52.5 (32.0–88.0)^a,b^	0.001
ALT (IU/L)	45.0 (27.5–75.5)^a^	60.0 (32.0–136.0)^a^	63.0 (37.5–87.0)^a^	0.04
Total bilirubin (mg/dL)	0.8 (0.6–1.4)^a^	1.1 (0.7–2.4)^b^	1.0 (0.6–2.2)^a,b^	0.02
Hemoglobin (g/dL)	13.5 (12.2–14.5)	13.2 (11.3–14.2)	13.1 (11.6–14.6)	0.31
Platelets (×10^6/^mm^3^)	229.0 (149.0–300.0)	212.0 (147.5–281.0)	219.0 (148.5–302.5)	0.52
INR	1.0 (1.0–1.2)	1.0 (1.0–1.2)	1.0 (1.0–1.1)	0.70
ERCP planned, n (%)	5 (4)	15 (10)	5 (6)	0.09

*Note*: Groups with different superscript letters differ with a *p*-value < 0.05 on the post-hoc Dunn test.

^1^ Based on the first abnormal cholangiogram.

^2^ Based on the first abnormal liver biochemistry.

^3^ Cirrhosis is defined by cirrhotic morphology on imagine, TE >14.4 kPa, MRE >4.93 kPa, APRI >2, or reported cirrhosis with stage 4.

^4^ Presence of ascites, variceal hemorrhage, and/or hepatic encephalopathy.

Abbreviations: AIH, autoimmune hepatitis; APRI, aspartate aminotransferase to platelet ratio index; ERCP, endoscopic retrograde cholangiopancreatography; IBD, inflammatory bowel disease; INR, international normalized ratio; MRE, magnetic resonance elastography; PSC, primary sclerosing cholangitis; TE, transient elastography; UDCA, ursodeoxycholic acid.

### Treatment of itch and medications of interest

Among 1120 itch-related medical encounters, antipruritic medication use was prescribed at 813 (73%), and endoscopic retrograde cholangiopancreatography (ERCP) was planned at 74 (7%) encounters with no association between planned ERCP and severity of pruritus (Table 1). Bile acid–binding resins (cholestyramine, colesevelam, or colestipol) were the most prescribed antipruritic medications (22% of the total cohort; Table 3). Roughly one-third (36%) of patients with any itch used a bile acid–binding resin, compared to 7% without an itch-related encounter. Other antipruritic medications used among patients with any itch included hydroxyzine (23%), rifampin (11%), sertraline (10%), and naltrexone (3%). The proportion of patients in the cohort prescribed UDCA was higher among those with any itch compared to no itch (74% vs. 62%, respectively, *p*<0.0001) and among those with moderate or severe itch (81% and 78%, respectively) compared to those with mild itch (65%; *p*=0.004)). However, the dose of UDCA was higher in patients with mild itch compared to those with moderate or severe itch (*p*=0.02) (Table 2).

**TABLE 3:** Frequency of antipruritic medications^a^

				Itch severity (n=359)^b^			
Medication	No itch (n=365), n (%)	Documented itch (n=359), n (%)	*p*	Mild (n=142), n (%)	Moderate (n=140), n (%)	Severe (n=77), n (%)	*p*
Bile acid–binding resin	26 (7)	131 (36)	<0.0001	34 (24)	61 (44)	36 (47)	0.0003
Hydroxyzine	20 (5)	81 (23)	<0.0001	26 (18)	29 (21)	26 (34)	0.03
Rifampin	4 (1)	41 (11)	<0.0001	4 (3)	16 (11)	21 (27)	<0.0001
Gabapentin	44 (12)	36 (10)	0.38	13 (9)	13 (9)	10 (13)	0.62
Sertraline	22 (6)	35 (10)	0.06	8 (6)	15 (11)	12 (16)	0.05
Doxepin	7 (2)	26 (7)	0.001	6 (4)	7 (5)	13 (17)	0.001
Fenofibrate	10 (3)	16 (4)	0.21	6 (4)	6 (4)	4 (5)	0.94
Naltrexone	2 (1)	12 (3)	0.01	1 (1)	5 (3)	6 (8)	0.02

^a^ Number of patients with a medication ever used.

^b^ Among patients with documented itch.

**FIGURE 2:**
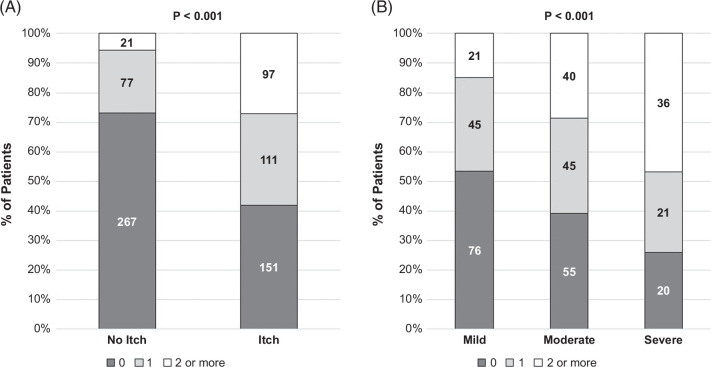
Number of antipruritic medications among patients with no itch versus any itch (A) and by severity of itch among patients with itch (B).

**FIGURE 3:**
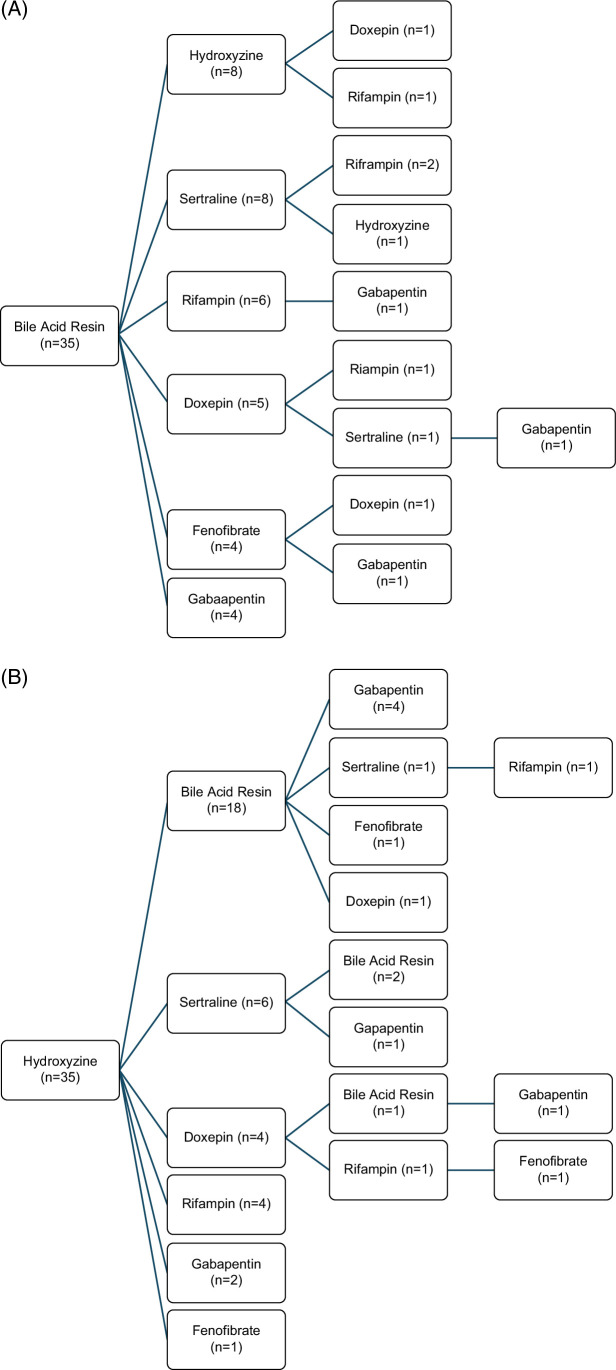
Sequence of therapy in patients with 2 or more antipruritic medications initially treated with a bile acid–binding resin (A) or hydroxyzine (B).

Overall, 306 (42%) patients had at least one prescription for an antipruritic medication, and 118 patients had 2 or more (Figure 2). Among those with 2 or more antipruritic medication prescriptions, 18 had a start date of 2 or more medications on the same date. The most common medications used in the 100 patients treated with sequential medications were bile acid–binding resins and hydroxyzine (Figure 3 and Supplemental Table S1, http://links.lww.com/HC9/B967). There was no consistency in second therapies following bile acid–binding resins, whereas bile acid–binding resins were the most common second treatment used in patients initially treated with hydroxyzine.

Larger proportions of patients with moderate (29%) or severe itch (47%) were more likely to have ever been prescribed ≥2 antipruritic medications compared to those with mild itch (15%%; *p*<0.0001). The most frequently prescribed medications for the treatment of IBD in this cohort were mesalamine (31%), sulfasalazine (9%), vedolizumab (8%), and infliximab (7%). No associations were observed between IBD medication use and itch (Supplemental Table S2, http://links.lww.com/HC9/B967).

## DISCUSSION

In this large retrospective, multicenter study of the prevalence, characteristics, and management of pruritus in the medical records of 724 patients with PSC, we found that itch was common and multiple antipruritic medications were frequently prescribed. The itch was noted in the medical records of nearly half of all included PSC patients, and roughly one-third of patients had itch categorized as moderate to severe based upon the providers’ notations. Those with moderate-to-severe itch had evidence of greater cholestasis on liver biochemistries and were more frequently prescribed antipruritic medications.

The prevalence of itch within our sample of 724 patients (49.6%) is consistent with previously observed rates of pruritus in other retrospective studies of patients with PSC,^20^^,^^21^^,^^24^ but less than that of self-reported itch.^24^ In addition, over half of those with an itch-related encounter were graded as moderate-to-severe itch—and nearly half of patients with severe pruritus were prescribed 2 or more antipruritic medications. These results suggest underreporting of itch in the medical record and, more importantly, reflect the lack of effective therapies for cholestatic pruritus.

Current guidance statements recommend a stepwise approach to treating itch in patients with PSC, but the lack of an effective treatment specific to PSC has led to differences in recommended medications between the American Association for the Study of Liver Disease (AASLD) and the European Association for the Study of the Liver (EASL).^1^^,^^11^ AASLD guidance recommends starting with bile acid–binding resins, followed by second-line therapies that include rifampin, sertraline, and naltrexone.^1^ In contrast, EASL guidelines recommend starting with bezafibrate, based upon emerging evidence of the effectiveness of fibrates, particularly bezafibrate, for the treatment of cholestatic pruritus in patients with PSC.^14^^,^^25^ Antihistamines are not recommended due to the lack of evidence that cholestatic itch is histamine-mediated or that antihistamines are effective.^26^ In the present study, we found that bile acid–binding resins were the most commonly used antipruritic medication but were prescribed for less than half of patients with moderate or severe pruritus and that antihistamines were used nearly as frequently. Rifampin was the most prescribed of the recommended second-line agents but was used in less than a quarter of patients with moderate or severe itch. Although bezafibrate is not available in the United States, another PPAR agonist, fenofibrate, was prescribed only rarely and at the same rate among patients without and without itch, suggesting that it was rarely, if ever prescribed to address pruritus.

Our results likely reflect the inadequacy of current therapies for cholestatic pruritus and how chronic itch impacts patients with PSC. Fortunately, there are several novel therapies in development that may offer better options for these patients. In addition to fibrates, novel PPAR agonists have demonstrated reductions in symptoms of itch in patients with PBC.^8^^,^^27^ Likewise, inhibitors of the ileal bile acid transporter (IBAT) have suggested they may have efficacy for the relief of itch in patients with PBC,^28^ PSC,^29^ and other forms of cholestatic itch.^30^

Strengths of our analysis include the large sample size recruited across 6 US health systems, and a diverse and well-characterized patient population, which enhances the generalizability of our findings. Our analysis also has some limitations. Incomplete data is unavoidable in any retrospective, observational analysis of “real world” medical records data. Likewise, our data is derived from nonstructured provider reports, which lack standardization and may not fully capture the patient experience of itch. As a result, our estimate of the prevalence of itch in patients with PSC is likely lower than the true prevalence. In addition, the period of observation was variable, which may have affected the number of visits and medications used. Further, over-the-counter medications were not captured. Finally, we could not ascertain if medications were used concurrently in an add-on approach or switched. To address these limitations, the ongoing prospective longitudinal arms of ItCh-PSC are collecting patient-reported itch with validated instruments and comprehensive medication reconciliation, which will allow us to better characterize patients’ experiences and medical needs.

In conclusion, this retrospective part of the ItCh-PSC study demonstrates that pruritus is a common symptom in PSC, and patients with moderate-to-severe pruritus are frequently prescribed several antipruritic medications. Novel and effective antipruritic medications are needed to relieve patients of this common and debilitating symptom of PSC.

## Supplementary Material
