# Supplementary material for: Treatment of pruritus in primary sclerosing cholangitis: Analysis of the consortium for autoimmune liver disease registry
Source: Hepatol Commun. 2025 May 6;9(5):e0703. doi: 10.1097/HC9.0000000000000703 (PMC12055165; doi:10.1097/HC9.0000000000000703)
Supplement: Supplementary file 1 [file hc9-9-e0703-s001.docx]

**Table S1.** Sequence of anti-pruritic medications in patients with 2 or more anti-pruritic medications

| First Medication | Second Medication | Third Medication | Fourth Medication | Fifth Medication |  |
| --- | --- | --- | --- | --- | --- |
| Bile Acid Resin (n= 35) | Doxepin n = 5 | Rifampin n=1  Sertraline n=1^a^ | Gabapentin n=1^a^ |  |  |
|  | Fenofibrate n = 4 | Doxepin n =1  Gabapentin n =1 |  |  |  |
|  | Gabapentin n = 4 |  |  |  |  |
|  | Hydroxyzine n = 8 | Doxepin n=1  Rifampin n =1 |  |  |  |
|  | Rifampin n = 6 | Gabapentin n =1 |  |  |  |
|  | Sertraline n = 8 | Rifampin n=2  Hydroxyzine n =1 |  |  |  |
|  | | | |  |  |
| Hydroxyzine (n= 35) | Bile Acid Resin n= 18 | Sertraline n = 1^a^  Fenofibrate n =1  Doxepin n = 1  Gabapentin n = 4 | Rifampin n = 1^a^ |  |  |
|  | Doxepin n = 4 | Rifampin n = 1^b^  Bile Acid Resin n = 1^c^ | Fenofibrate n = 1^b^  Gabapentin n =1^c^ |  |  |
|  | Fenofibrate n = 1 |  |  |  |  |
|  | Gabapentin n = 2 |  |  |  |  |
|  | Rifampin n = 4 |  |  |  |  |
|  | Sertraline n = 6 | Bile Acid Resin n = 2  Gabapentin n =1 |  |  |  |
|  | | | | |  |
| Gabapentin (n = 9) | Bile Acid Resin n = 2 | Hydroxyzine n = 1^a^ | Rifampin n =1^a^ | Fenofibrate n = 1^a^ | |
|  | Doxepin n = 4 | Hydroxyzine n = 1  Fenofibrate n = 1 |  |  | |
|  | Hydroxyzine n = 1 | Doxepin n = 1^b^ | Bile Acid Resin n =1^b^ |  | |
|  | Rifampin n = 2 | Sertraline n = 1^c^ | Doxepin n =1^c^ | Hydroxyzine n = 1^c^ | |
|  | | | | | |
| Sertraline (n= 9) | Bile Acid Resin n = 5 | Gabapentin n = 2^a^  Rifampin n = 1^b^ | Hydroxyzine n =1^a^  Hydroxyzine n =1^b^ |  | |
|  | Gabapentin n = 2 | Bile Acid Resin n =1 |  |  | |
|  | Hydroxyzine n = 2 | Bile Acid Resin n = 1^c^ | Rifampin n = 1^c^ | Gabapentin n = 1^c^ | |
|  | | | | | |
| Rifampin (n= 7) | Bile Acid Resin n = 2 |  |  |  | |
|  | Doxepin n = 3 | Bile Acid Resin n =2^a^ | Sertraline n =1^a^ |  | |
|  | Gabapentin n = 1 |  |  |  | |
|  | Sertraline n = 1 |  |  |  | |
|  | | | | | |
| Fenofibrate (n= 4) | Bile Acid Resin n = 2 |  |  |  | |
|  | Gabapentin n = 1 |  |  |  | |
|  | Sertraline n = 1^a^ | Bile Acid Resin n = 1^a^ |  |  | |
|  | | | | | |
| Doxepin (n= 1) | Hydroxyzine n = 1 |  |  |  | |

Superscripts denote individual patients who switched medications during the study period.

**Table S2.** Medication use for inflammatory bowel disease.

| Medication | No Itch (n=365) | Mild (n=142) | Mod (n=140) | Severe (n=77) |
| --- | --- | --- | --- | --- |
| Mesalamine (*g*) | 103 (28%) | 42 (30%) | 55 (39%) | 25 (32%) |
| Sulfasalazine (*g*) | 33 (9%) | 8 (6%) | 13 (9%) | 11 (14%) |
| Adalimumab (*mg*) | 17 (5%) | 12 (8%) | 10 (7%) | 8 (10%) |
| Certolizumab (*mg*) | 2 (1%) | - | 2 (1%) | 1 (1%) |
| Infliximab (*mg*) | 21 (6%) | 8 (6%) | 15 (11%) | 6 (8%) |
| Ustekinumab (*mg*) | 11 (3%) | 11 (8%) | 3 (2%) | 4 (5%) |
| Vedolizumab (*mg*) | 32 (9%) | 10 (7%) | 4 (3%) | 10 (13%) |
